# Face-off Droop: A Case Report of Pediatric Stroke

**DOI:** 10.5811/cpcem.6586

**Published:** 2024-04-24

**Authors:** Duncan Robertson, Hayden F. Peirce, Marek D. Nicpon, Eric M. Otterson, Laurel O’Connor, Julia G. Rissmiller, Zachary W. Binder

**Affiliations:** * University of Massachusetts, Chan Medical School, Department of Emergency Medicine, Worcester, Massachusetts; † University of Massachusetts, Chan Medical School, Department of Pediatrics, Worcester, Massachusetts; ‡ University of Massachusetts, Chan Medical School, Department of Radiology, Worcester, Massachusetts

**Keywords:** *stroke*, *clavicle fracture*, *pseudoaneurysm*

## Abstract

**Introduction:**

Cerebrovascular accidents rarely occur in children; the incidence of ischemic stroke in patients <16 years of age is between 0.6–7.9/100,000. However, they are the fourth most common cause of acute neurological deficits in the pediatric population, and possible cases should be evaluated with a high index of suspicion to ensure timely intervention.

**Case Report:**

We describe a previously healthy 17-year-old male who presented to the pediatric emergency department with a left facial droop and hemiparesis consistent with a stroke. The patient’s age and lack of comorbidities made this an extremely uncommon presentation. Our patient’s neurologic symptoms were believed to have been caused by a recent traumatic clavicular injury sustained two weeks prior, which subsequently led to vascular insult.

**Conclusion:**

Cerebrovascular accidents are an important cause of morbidity and mortality in pediatric patients. Cerebrovascular accidents in children are most often secondary to congenital causes; however, care should be taken to assess for acquired causes, such as trauma to major blood vessels. While rarely implicated in traumatic injuries, arterial structures posterior to the medial clavicle can result in severe complications.

Population Health Research Capsule What do we already know about this clinical entity?

*Cerebrovascular accident (CVA) is a major cause of morbidity and mortality for patients of all ages and can arise from many different etiologies.*

What makes this presentation of disease reportable?

*Our young patient had no risk factors for stroke except for an occult injury that arose from prior trauma. This initially confounded the cause during initial presentation.*

What is the major learning point?

*Although CVA is relatively rare in the pediatric population, it must remain on the differential as remote traumatic injury may create a nidus for thromboembolism.*

How might this improve emergency medicine practice?

*Expedient care of CVA in the ED is dependent on quick recognition and must be considered even in those with few perceived risk factors.*

## INTRODUCTION

While more common in older adults, strokes, or cerebrovascular accidents (CVA), rarely occur in children. The incidence of ischemic stroke in children <16 years of age is between 0.6–7.9/100,000, and in young adults <45 years old is between 8–100/100,000 per year.^1^^,^^2^ The traditional risk factors for stroke—hypertension, smoking, diabetes, and hypercholesterolemia—are less prevalent in pediatric patients. In children, CVAs are more commonly associated with cardiac conditions, hematologic conditions, vasculopathies, and metabolic disorders.^1^ Pregnancy, exogenous hormone use, smoking, illicit drug use, and premature atherosclerosis can increase the risk of CVA in young adults.^1^ We present the case of a healthy adolescent male who presented to the pediatric emergency department (PED) with a stroke despite having no identifiable risk factors. Written permission from the patient’s guardians and assent from the patient were obtained to present this case.

## CASE REPORT

Emergency medical services (EMS) responded to a hockey rink for a chief complaint of “neck injury.” Paramedics encountered a 17-year-old male lying supine on the locker room floor. The patient’s trainer reported that the patient had collapsed on the ice and could not stand. The patient complained of light-headedness and was noted to have left-sided facial droop and paralysis of his left upper extremity. He denied headache or neck pain. There was no reported traumatic injury on the day of presentation. The patient denied medical or surgical history, drug use, or alcohol use. A video of the incident was obtained (Video 1).

The exam performed by EMS was notable for tachycardia, left-sided facial droop, and absent strength of the left shoulder, elbow, forearm, hand, and fingers. Spinal precautions were applied, and EMS bypassed a community hospital in favor of the regional pediatric trauma center. Upon arrival to the PED, the patient was activated as a trauma. On assessment in the resuscitation bay, a persistent left lower facial droop, a Glasgow Coma Scale of 15, and a negative extended focused assessment with sonography in trauma was present. No external signs of trauma were noted on the exam.

**Image 1.**
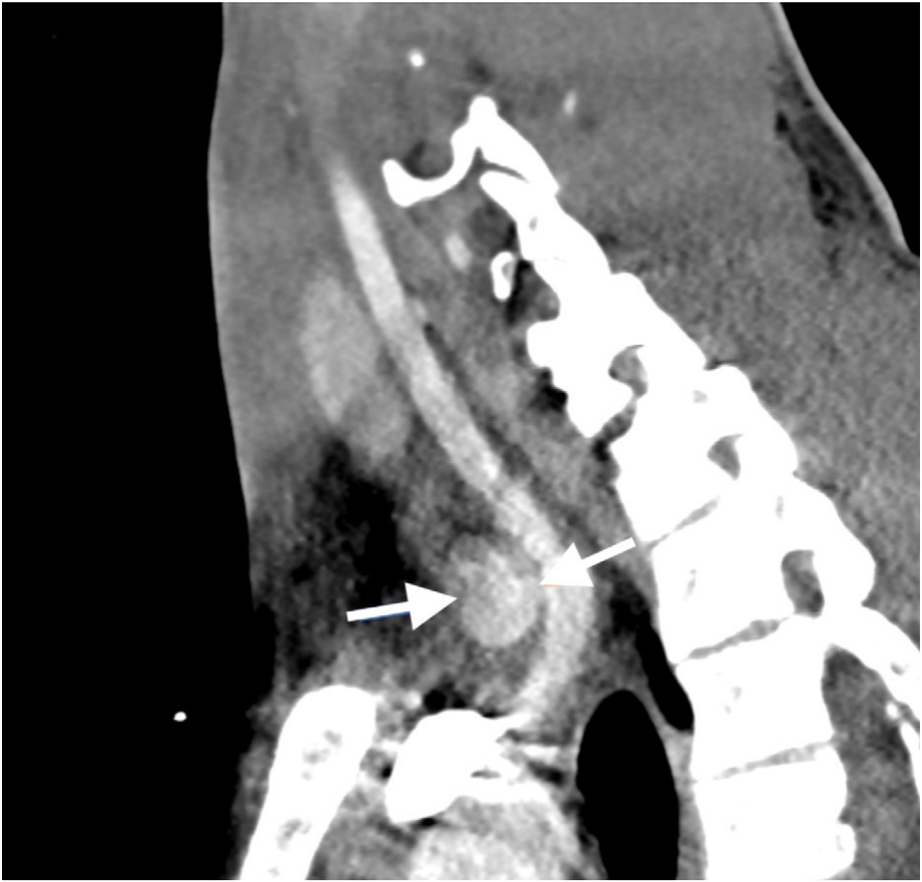
Sagittal multiplanar reconstruction of the computed tomography angiogram of the head and neck demonstrates a 1.8 cm amorphous, hyperdense, oval structure abutting the anterior aspect of the junction of the brachiocephalic and right common carotid arteries, with an apparent connecting neck extending from the brachiocephalic artery (arrows), suggesting a pseudoaneurysm or contained rupture.

At that time the patient was identified as a suspected stroke with a National Institute of Health Stroke Scale score of three. Pediatric neurology was consulted, and the patient was emergently taken for computed tomography (CT) head, CT cervical spine, and CT angiogram (CTA) of the head and neck. Computed tomography did not demonstrate any acute intracranial abnormality or fracture of the cervical spine. The CTA of the head and neck showed “a 1.8 cm amorphous hyperdensity abutting the anterior aspect of the junction of the brachiocephalic and right common carotid arteries with an apparent neck extending from the brachiocephalic artery, suggesting pseudoaneurysm or contained rupture” (Image 1).

**Image 2.**
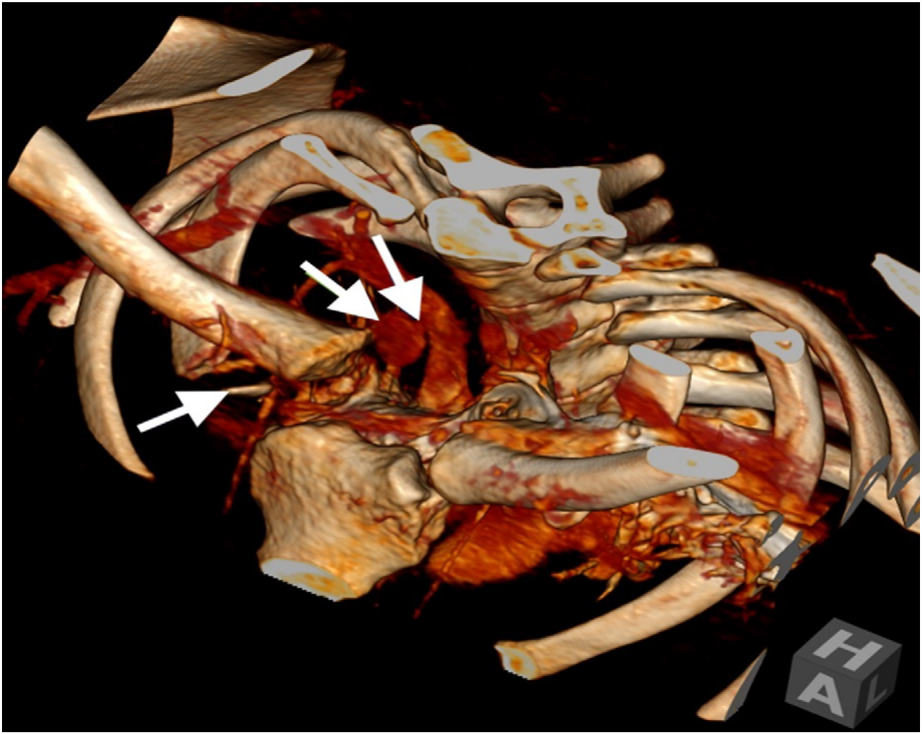
Three-dimensional reconstruction of the thoracic inlet using the computed tomography angiogram of the head and neck demonstrates fracture of the right clavicular head with an anterior fracture fragment posterior displacement of the clavicle with abutment of the pseudoaneurysm/contained rupture (arrows). The connecting neck extending from the brachiocephalic artery is also depicted.

A non-occlusive filling defect in the proximal right subclavian artery was suggestive of thrombus and raised concern for an embolic process. Decreased flow in the right distal second segment and third segments of the right vertebral artery supported a concern for a thrombus. There were no large vessel occlusions or significant stenoses of the major intracranial arteries. Finally, a Salter-Harris I fracture of the right clavicular head with surrounding contusion/hematoma was noted, along with dislocation of the sternoclavicular joint. The posteriorly displaced clavicle was noted to abut the pseudoaneurysm/contained rupture (Image 2).

After these findings were made radiographically, the patient was asked specifically about injury to his right upper chest. He reported that two weeks earlier he had sustained a blunt injury to the area during hockey practice. He had been evaluated by his school trainer, and radiographs had been obtained that were interpreted as negative. He had continued to play hockey over the subsequent two weeks with moderate but improving pain. As part of the patient’s trauma evaluation, a chest radiograph was performed, which demonstrated left tracheal deviation, likely due to the clavicle fracture. Laboratory data revealed normal coagulation studies and lipid profile.

Once the subclavian artery was identified as an apparent thrombotic source, consultations were placed to vascular surgery and cardiothoracic surgery. The patient was taken emergently to the operating room for repair of the right innominate artery pseudoaneurysm via sternotomy. Additionally, an embolectomy of the right subclavian artery was performed. The patient awoke post-operatively with a strong right radial pulse and neurologically intact.

**Image 3.**
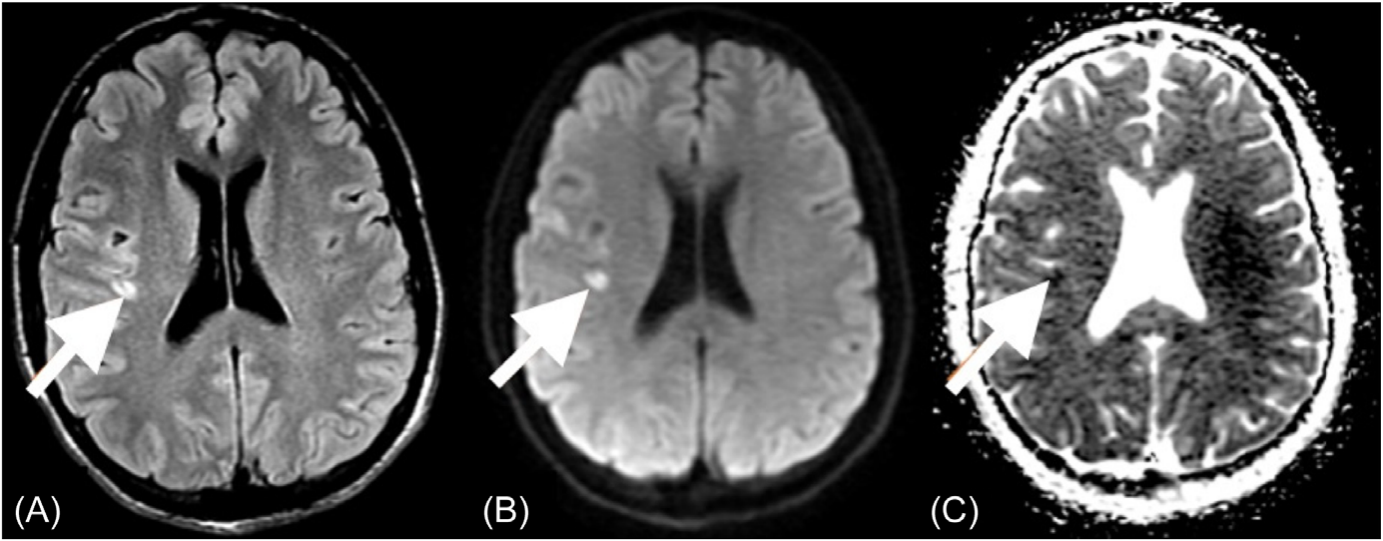
Non-contrast brain magnetic resonance imaging demonstrates a hyperintense lesion on the axial T2- weighted image in the posterior right insular cortex (A) with restricted diffusion as demonstrated by focus of hyperintense signal on the diffusion weighted image (B), and a focus of hypointense signal on the apparent diffusion coefficient map (C), consistent with an acute infarct. Similar lesions were also identified in the anterior right frontal lobe and anterior aspect of the right parietal lobe.

Magnetic resonance imaging (MRI) performed the following day revealed several small acute infarcts to the right frontal lobe, the posterior margin of the right insular cortex (Image 3), and the right parietal lobe. These findings support the hypothesis that an embolic process caused our patient’s presenting neurologic symptoms. He was placed on aspirin (81 milligrams daily for three months) and was discharged neurologically intact on postoperative day three with plans for vascular surgery follow-up and interval repair of the clavicular dislocation by orthopedic surgery.

## DISCUSSION

This case describes a 17-year-old male who presented to the PED with weakness of his left face and arm. Imaging revealed a pseudoaneurysm of the brachiocephalic artery likely due to a right clavicular fracture. The pseudoaneurysm was repaired, and the patient was discharged from the hospital without neurological deficits. There are several noteworthy considerations in this case. First, CVA is rare in children, especially in those without medical comorbidities.^1^^,^^2^ Older patients experiencing a CVA tend to present with signs of aphasia, visual disturbance, and hemiparesis. In contrast, pediatric patients may present with altered mental status, lethargy, and seizures.^3^^,^^4^

Pediatric stroke secondary to cardiac disease is associated with bilateral deficits, anterior and posterior circulation involvement, and seizures.^4^ Conversely, stroke secondary to trauma or embolic phenomenon is associated with posterior circulation involvement, and is more likely to present with dysarthria, hemiparesis, visual field defect, or ataxia. ^5^ While rare, stroke is the fourth most common cause of acute focal neurological deficit in children after hemiplegic migraine, seizure, and Bell’s palsy.^6^ Paramount to the evaluation of stroke is imaging. Diffusion-weighted MRI coupled with vascular MR angiography is the gold standard in the evaluation of ischemic stroke.^7^^,^^8^ If unavailable within the first hour of presentation, guidelines recommend non-contrast CT and CTA of the head and neck.^8^ While radiation is a factor to consider in the evaluation of pediatric patients, the benefits of CT imaging in this circumstance outweigh potential harm.

The management of pediatric stroke requires a multidisciplinary approach involving the ED, neurology, pharmacy, the patient, and the patient’s family. Antiepileptics should be initiated if seizures are present.^7^^–^^10^ Treatment of acute ischemic stroke with tissue plasminogen activator (tPA) or endovascular intervention have shown benefits in adults; however, these therapies remain controversial in children. A joint statement by the American Heart Association/American Stroke Association recommends that tPA or endovascular intervention be considered in pediatric patients who have radiographically confirmed large artery occlusion and persistent disabling neurological deficits, in consultation with neurology and endovascular surgery.^10^

It is suspected that the pseudoaneurysm and subsequent emboli formation in our patient’s subclavian artery were the most likely cause of his CVA. Two potential mechanisms have been suggested: 1) direct embolization from the subclavian artery clot into the carotid artery; or 2) migration of the brachiocephalic artery clot into the subclavian artery with smaller emboli then transiting through the carotid artery.

Another remarkable aspect of this case was the profound sequela of the patient’s clavicular injury. His neurologic symptoms ultimately arose from a clavicle fracture that had occurred two weeks prior. We suspect that the fracture caused the initial insult to the brachiocephalic artery, which then led to the formation of the pseudoaneurysm. Clavicle fractures are managed based on their location, angulation, and the degree of compromise to surrounding tissue. Distal and midshaft clavicular fractures are typically managed conservatively with a sling and orthopedic surgery follow-up.^11^ Fractures of the medial clavicle, such as the one sustained by our patient, are rare, representing 2–6% of all clavicle fractures.^12^^,^^13^

The medial clavicular physis closes between 22–25 years of age. As a result, fractures of the medial clavicle most often occur in patients <25 years old.^14^ These fractures are associated with injuries to vascular structures within the mediastinum such as the brachiocephalic artery, aorta, and subclavian artery.^14^ Attention must be taken to ensure that these vascular structures are not injured when evaluating medial clavicular fractures.^15^ A review of the literature yielded a single report of a similar incident where a traumatic clavicular injury caused a secondary CVA in a young adult; however, there is little available data on the incidence of CVA caused by blunt traumatic injury in pediatric or adult patients.^16^

Finally, early and effective communication between teams can minimize delays in the assessment and management of patients with time-sensitive and/or uncommon ED presentations. In this case, EMS diverted to a pediatric trauma center and the multidisciplinary team was present on arrival. Once acute trauma was deemed less likely, the patient was identified as a suspected stroke victim, which resulted in expedited neurological evaluation. This identification of a stroke may not have happened as rapidly without a conscientious assessment by EMS and frontline paramedics. Any delay could have resulted in permanent neurologic injury. Our patient likely benefitted from being transported directly to a medical center with the appropriate resources to treat his rare presentation.

## CONCLUSION

While rarer than in adults, CVAs are an important cause of morbidity and mortality in pediatric patients. Cerebrovascular accidents in children are most often secondary to congenital causes; however, care should be taken to assess for acquired causes, as occurred in our patient. The region posterior to the medial clavicle, while rarely implicated in traumatic injury, holds many important arterial structures. As a result, injuries to this area should be evaluated with a high index of suspicion. Finally, when pediatric stroke is being considered it is important to use all available resources to obtain prompt imaging and expedite evaluation to increase the chances of a favorable outcome.

## Supplementary Information

Video 1. The patient (#22) can be seen initially at the right of the video. As the camera pans toward the right, the patient is seen at the center. As he skates backward he falls. On multiple attempts to get back up, it appears his left leg and arm have decreased coordination and weakness. He is escorted off the ice to be evaluated by medical personnel.
